# Supplementary material for: Investigating microRNA Profiles in Prostate Cancer Bone Metastases and Functional Effects of microRNA-23c and microRNA-4328
Source: Cancers (Basel). 2023 Apr 24;15(9):2437. doi: 10.3390/cancers15092437 (PMC10177411; doi:10.3390/cancers15092437)
Supplement: Supplementary file 1 [file cancers-15-02437-s001.zip › Supplementary Figure_S1.pdf]

Figure S1.

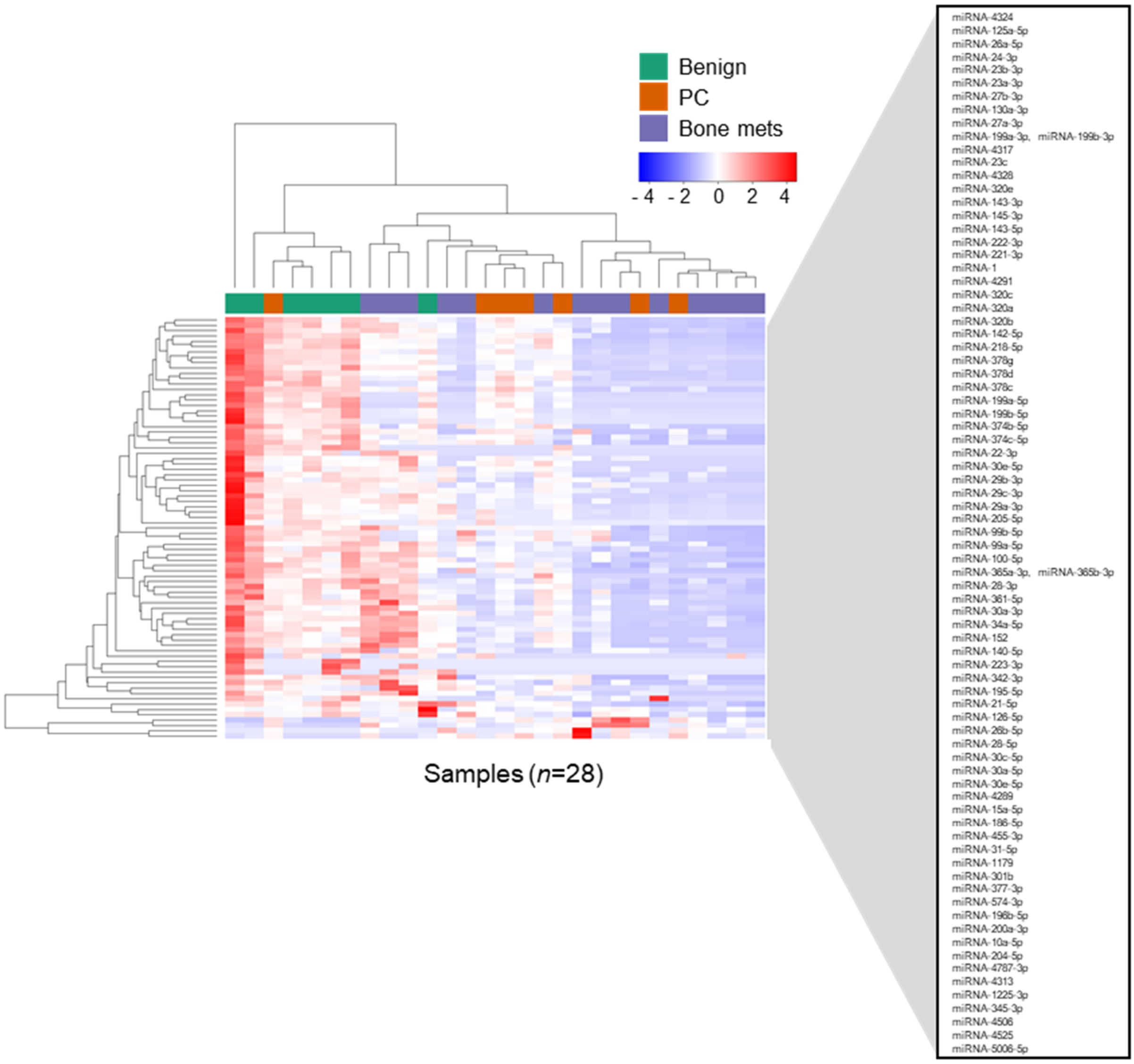

Figure S1. Differentially expressed microRNAs (miRNAs) in bone metastatic prostate cancer. Heatmap demonstrating unsupervised hierarchical clustering of biopsies (*n* = 28) and microRNAs (miRNAs) (*n* = 79) differing significantly ($p < 0.05$) in castration resistant prostate cancer bone metastases (bone mets) (*n* = 14) and localized prostate cancer (PC) (*n* = 7) as compared to benign prostate tissue (benign) (*n* = 7).
