# Supplementary material for: Investigating microRNA Profiles in Prostate Cancer Bone Metastases and Functional Effects of microRNA-23c and microRNA-4328
Source: Cancers (Basel). 2023 Apr 24;15(9):2437. doi: 10.3390/cancers15092437 (PMC10177411; doi:10.3390/cancers15092437)
Supplement: Supplementary file 1 [file cancers-15-02437-s001.zip › Supplementary Figure_S2.pdf]

Figure S2.

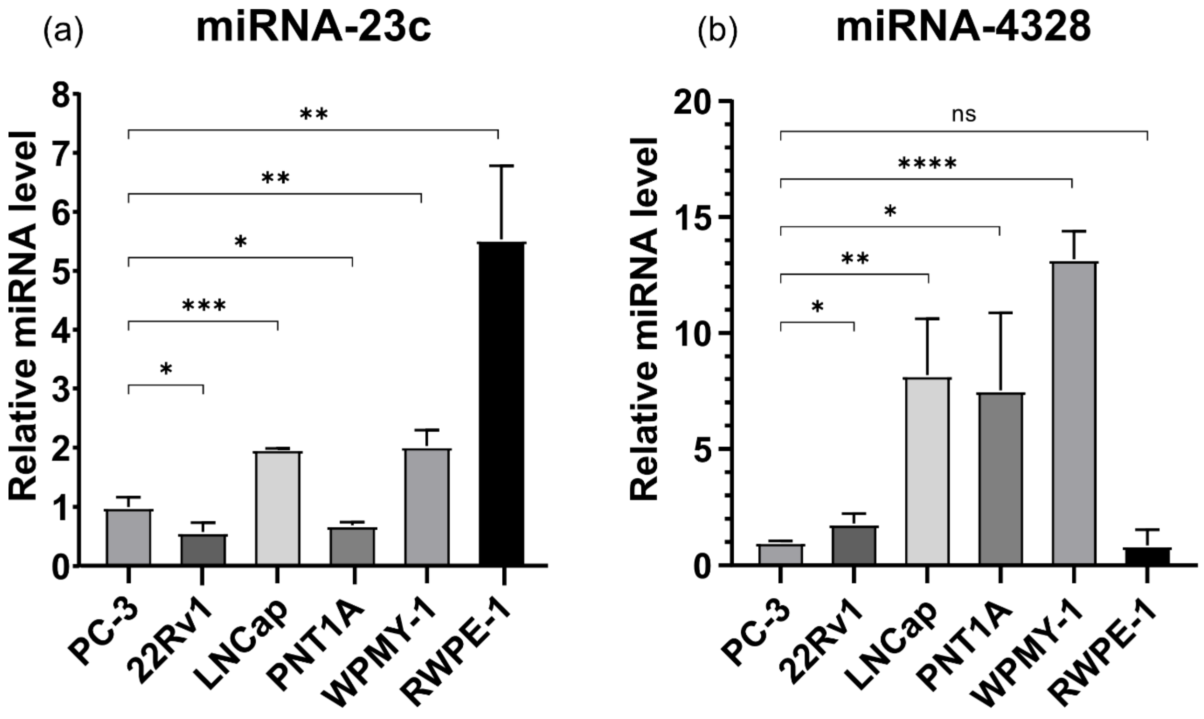

Figure S2. Endogenous levels of miRNA-23c and miRNA-4328 in different prostate and prostate cancer cell lines. Relative miRNA-23c (a) and miRNA-4328 (b) levels in the prostate cell lines RWPE-1, PNT1A, WPMY-1, and in the prostate cancer cell lines LNCaP, 22Rv1 and PC-3 are shown. Levels of miRNA were determined with RT-qPCR and are displayed in relation to levels in the PC-3 cell line (n=3 per cell line). *$p < 0.05$, **$p < 0.01$, ***$p < 0.001$, ****$p < 0.0001$, ns $p > 0.05$ (unpaired t-test).
