# Supplementary material for: Investigating microRNA Profiles in Prostate Cancer Bone Metastases and Functional Effects of microRNA-23c and microRNA-4328
Source: Cancers (Basel). 2023 Apr 24;15(9):2437. doi: 10.3390/cancers15092437 (PMC10177411; doi:10.3390/cancers15092437)
Supplement: Supplementary file 1 [file cancers-15-02437-s001.zip › Supplementary Figure_S3.pdf]

Figure S3.

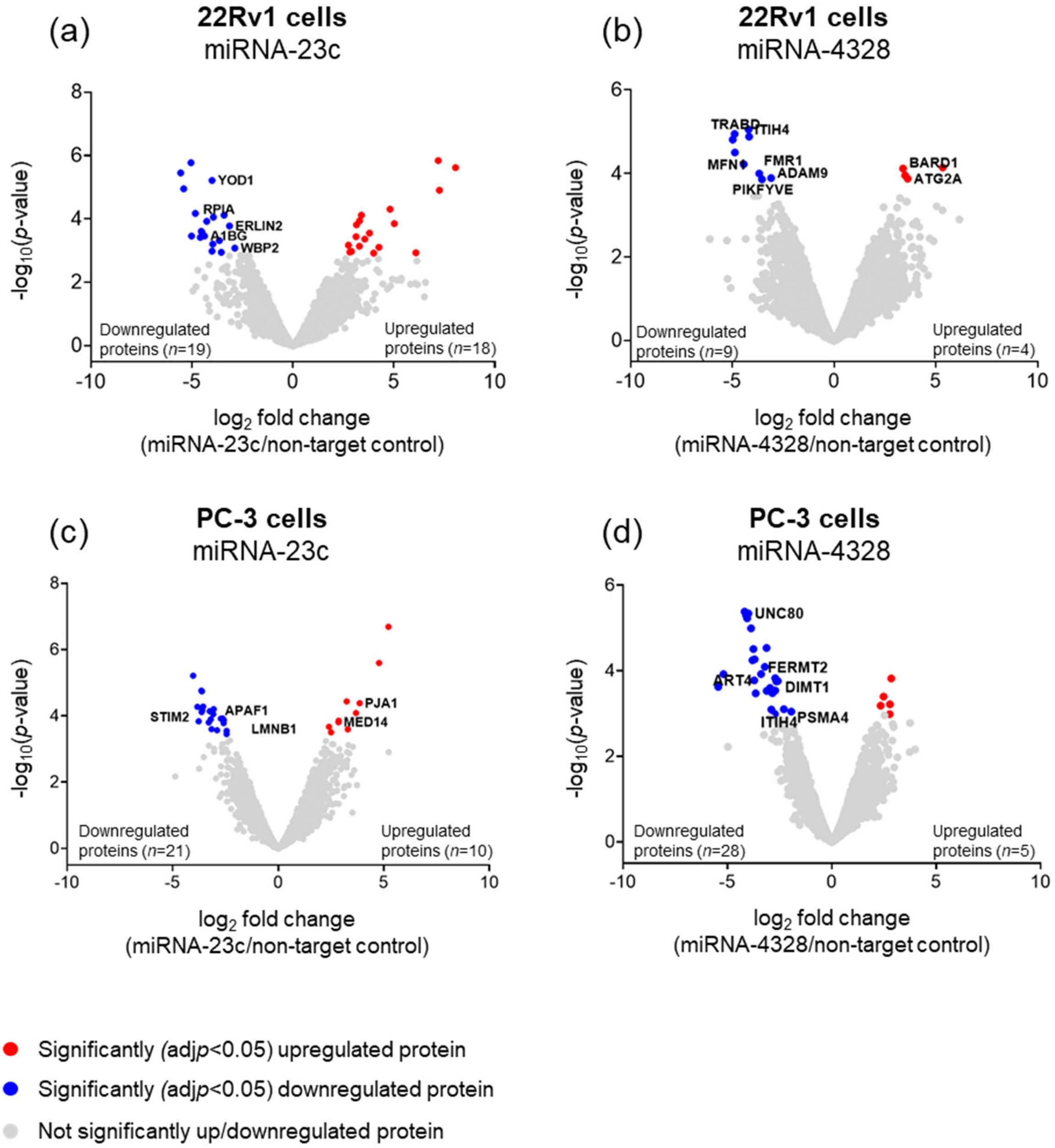

Figure S3. Volcano plots highlighting differentially expressed proteins following overexpression of miRNA-23c (a, c) or miRNA-4328 (b, d) in the 22Rv1 (a, b) and PC-3 (c, d) cell lines. Red and blue dots symbolize significantly up- and down-regulated proteins, respectively (adj$p$ < 0.05, n = 3 single cell clones per cell line). Abbreviations indicate proteins being predicted as targets for the miRNA in the TargetScan (targetscan.org) database.
