# Supplementary material for: Investigating microRNA Profiles in Prostate Cancer Bone Metastases and Functional Effects of microRNA-23c and microRNA-4328
Source: Cancers (Basel). 2023 Apr 24;15(9):2437. doi: 10.3390/cancers15092437 (PMC10177411; doi:10.3390/cancers15092437)
Supplement: Supplementary file 1 [file cancers-15-02437-s001.zip › Supplementary Figure_S4.pdf]

Figure S4.

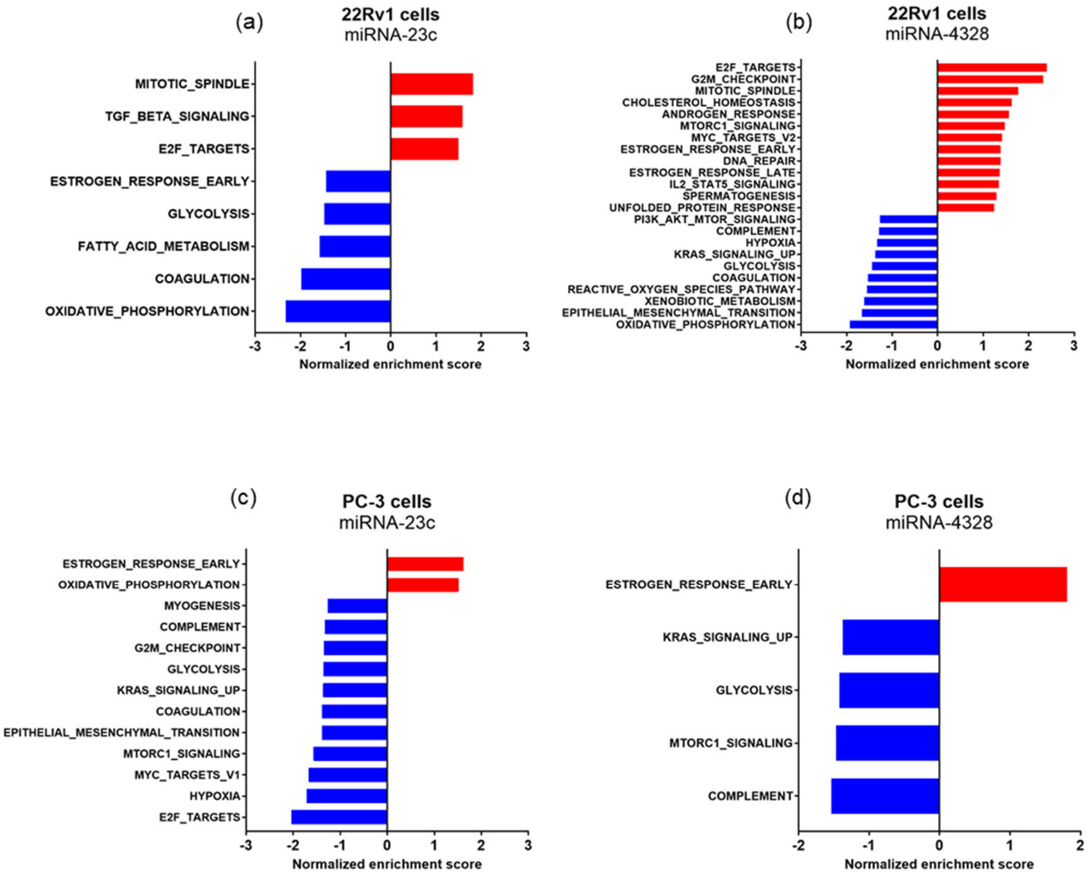

Figure S4. Gene set enrichment analysis highlighting predefined cancer hallmark gene sets significantly affected by overexpression of miRNA-23c (a, c) or miRNA-4328 (b, d) in the 22Rv1 (a, b) and PC-3 (c, d) cell lines. The normalized enrichment score displays whether genes in a gene set are over- or underrepresented at the top or bottom of the ranked list of all genes. Blue and red represent normalized enrichment scores that were significantly affected in a negative or positive way, respectively, according to analysis of single cell clones (n = 3 per cell line) (adj$p < 0.25$). The gene sets were retrieved from the Molecular Signatures Database v7.4 (www.gsea-msigdb.org).
