# Supplementary material for: Investigating microRNA Profiles in Prostate Cancer Bone Metastases and Functional Effects of microRNA-23c and microRNA-4328
Source: Cancers (Basel). 2023 Apr 24;15(9):2437. doi: 10.3390/cancers15092437 (PMC10177411; doi:10.3390/cancers15092437)
Supplement: Supplementary file 1 [file cancers-15-02437-s001.zip › Supplementary Figure_S5.pdf]

Figure S5.

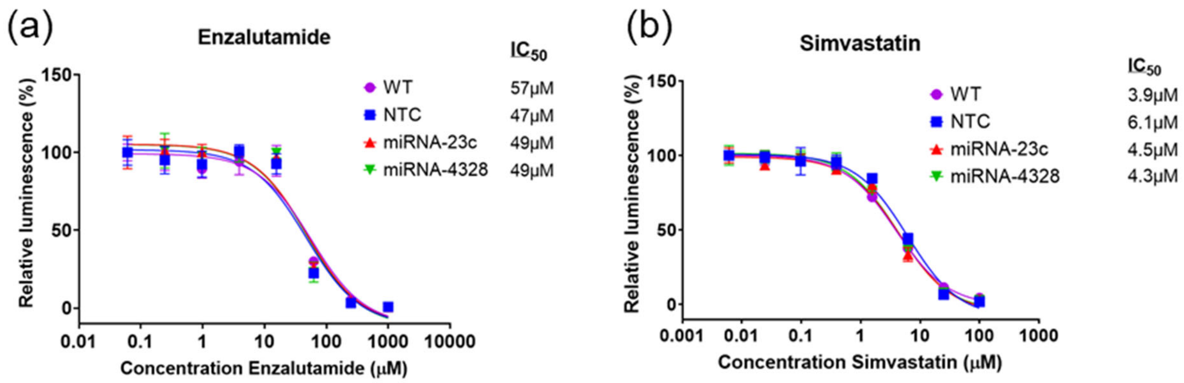

Figure S5. Cell response to Enzalutamide (a) and Simvastatin (b) after overexpression of miRNA-23c or miRNA-4328 in the 22Rv1 cell line. The half maximal inhibitory concentrations (IC_{50}) were determined by seeding cells in triplicates and growing them in 8 different drug concentrations over 96h. The CellTiter-Glo® Luminescent Cell Viability reagent was used to obtain the relative cell numbers at 96h, based on the quantity of ATP produced, analyzed as an indicator of metabolically active cells (CellTiter-Glo® Luminescent Cell Viability Assay).
