# Supplementary material for: Investigating microRNA Profiles in Prostate Cancer Bone Metastases and Functional Effects of microRNA-23c and microRNA-4328
Source: Cancers (Basel). 2023 Apr 24;15(9):2437. doi: 10.3390/cancers15092437 (PMC10177411; doi:10.3390/cancers15092437)
Supplement: Supplementary file 1 [file cancers-15-02437-s001.zip › Supplementary Figure_S6.pdf]

**Figure 6S.**

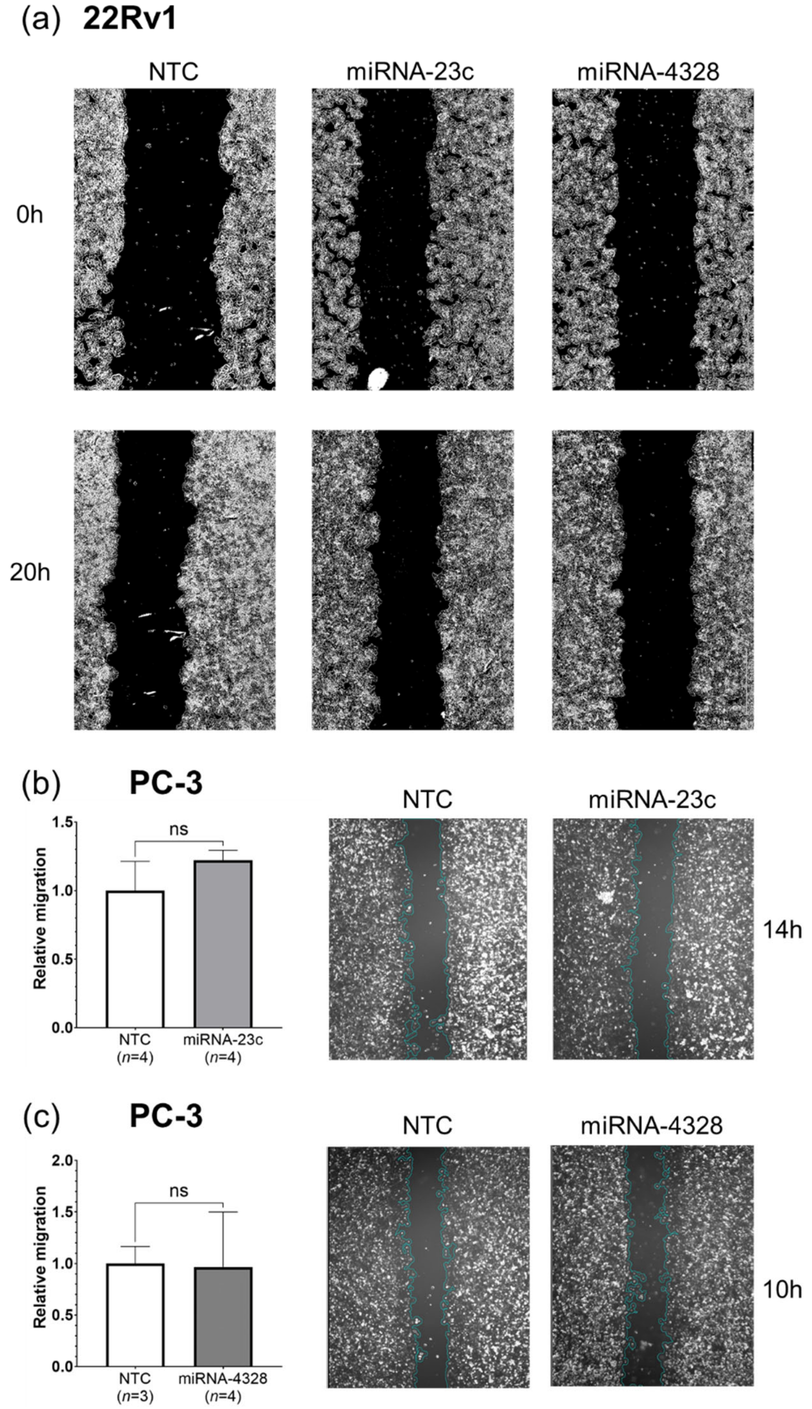


**Figure S6. Wound healing assay of 22Rv1 and PC-3 cells overexpressing miRNA-23c or miRNA-4328.** (a) Examples of wounds of 22Rv1 cells at baseline (0h) and after 20h. Examples of wounds of PC-3 cells overexpressing miRNA-23c (b) and miRNA-4328 (c). Graphs display the relative difference in wound area as compared to 0h. The wound area was quantified in Image J using a plugin for scratch wound healing assays. ns: $p > 0.05$, $n = 3\text{-}4$.
