# Supplementary material for: Investigating microRNA Profiles in Prostate Cancer Bone Metastases and Functional Effects of microRNA-23c and microRNA-4328
Source: Cancers (Basel). 2023 Apr 24;15(9):2437. doi: 10.3390/cancers15092437 (PMC10177411; doi:10.3390/cancers15092437)
Supplement: Supplementary file 1 [file cancers-15-02437-s001.zip › Supplementary Figure_S7.pdf]

Figure S7.

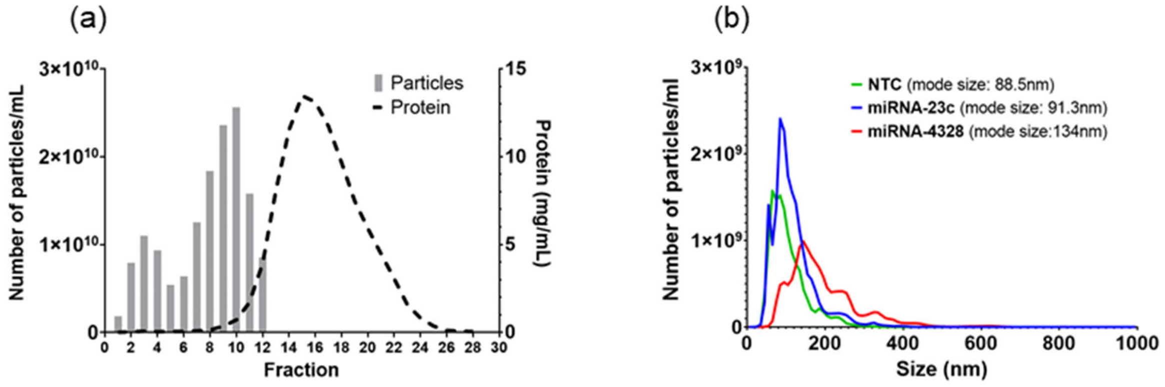

Figure S7. Particle distribution and protein concentration of sample fractions obtained by size-exclusion chromatography of conditioned cell culture medium from PC-3 cells following miRNA-23c or -4328 overexpression. (a) Protein concentrations for all fractions (*n* = 28) and particle concentrations for the first 12 fractions of conditioned cell culture medium from PC-3 cells with stable overexpression of miRNA-23c, shown as a representative example for the samples shown in B. (b) Particle concentration and size of pooled protein-free fractions (fractions 2-10) from PC-3 cells with stable overexpression of miRNA-23c, miRNA-4328, or NTC. Particles were analyzed by the nanoparticle tracking system NanoSight NS300 and proteins by A280 absorbance.
