# Supplementary material for: Investigating microRNA Profiles in Prostate Cancer Bone Metastases and Functional Effects of microRNA-23c and microRNA-4328
Source: Cancers (Basel). 2023 Apr 24;15(9):2437. doi: 10.3390/cancers15092437 (PMC10177411; doi:10.3390/cancers15092437)
Supplement: Supplementary file 1 [file cancers-15-02437-s001.zip › Supplementary methods.pdf]

Supplementary methods:

Relative protein quantification

Relative quantification was performed to compare protein expression in triplicates of PC3 and 22Rv1 cell clones overexpressing miRNA-23c and -4328, respectively, with protein expression in NTC cell clones (n=3). The samples were homogenized in lysis buffer (200 µl, 50 mM triethylammonium bicarbonate (TEAB), 2% sodium dodecyl sulfate) using FastPrep® (lysis matrix B, MP Biomedicals) and protein concentration was determined by Pierce™ BCA Protein Assay (Thermo Scientific). The samples and two references (representative pools from PC3 or 22Rv1 cell samples) were digested using modified filter-aided sample preparation (FASP) method (Wisniewski JR et. al. Nat Methods. 2009 May;6(5):359-62). In short, samples (13 µg) were reduced (100 mM dithiothreitol, 60°C, 30 min), transferred to cut-off filters (30 kDa MWCO Pall Nanosep, Sigma-Aldrich), washed several times with 8 M urea and once with digestion buffer (DB, 50 mM TEAB, 0.5% sodium deoxycholate (SDC)) prior to alkylation (10 mM methyl methanethiosulfonate in DB, RT, 30 min). Samples were digested with trypsin (Pierce MS grade Trypsin, Thermo Fisher Scientific, ratio 1:100) at 37°C overnight and an additional portion of trypsin was added and incubated for another two hours. Peptides were collected by centrifugation and labelled using 10-plex tandem mass tagging (TMT) (Thermo Fisher Scientific). The samples were combined into two TMT-set, SDC was removed by acidification with 10% TFA and desalted (Pierce peptide desalting spin columns, Thermo Fischer Scientific). The sets were fractionated into 10 fractions with basic reversed-phase chromatography (bRP-LC) using a Dionex Ultimate 3000 UPLC system (Thermo Fischer Scientific) and a reversed-phase XBridge BEH C18 column (3.5 µm, 3.0x150 mm, Waters Corporation) with a gradient from 3% to 100% acetonitrile in 10 mM ammonium formate at pH 10.00 over 23 min at a flow of 400 µL/min.

nanoLC-MS analysis and database search

Each fraction was analysed on Orbitrap Fusion™ Tribrid™ mass spectrometer interfaced with nLC1200 liquid chromatography system (both Thermo Fisher Scientific). Peptides were separated on an in-house constructed analytical column (350x0.075 mm I.D.) packed with 3 µm Reprosil-Pur C18-AQ particles (Dr Maisch, Germany) using a gradient from 3% to 80% acetonitrile in 0.2% formic acid over 85 min at a flow of 300 nL/min. Precursor ion mass spectra were acquired at 120 000 resolution and MS2 analysis was performed in a data-dependent multinotch mode where precursors were isolated in the quadrupole with a 0.7 m/z

isolation window. Peptides with charge states 2 to 7 were selected for fragmentation CID 35 and dynamic exclusion was set to 60 s and 10 ppm. The top 5 most abundant MS2 fragment ions were isolated simultaneous for fragmentation (MS3) by HCD 65 and detected in the Orbitrap at 50 000 resolutions.

The data files for each set were merged for identification and relative quantification using Proteome Discoverer version 2.4 (Thermo Fisher Scientific). The search was against *Homo Sapiens* (Swissprot Database Nov 2020) using Mascot 2.5 (Matrix Science) with MS peptide tolerance of 5 ppm and fragment ion tolerance of 0.6 Da. Tryptic peptides were accepted with one missed cleavage, variable modifications of methionine oxidation and fixed cysteine. Percolator was used for the peptide-spectrum match (PSM) validation with the strict false discovery rate (FDR) threshold of 1%. Samples were normalized on the total peptide amount. For protein quantification unique peptides at FDR threshold 1% with a minimum SPS 65 were taken into account. Quantified proteins were filtered at 5% FDR. The reference samples were used as denominator and for calculation of the ratios.
