# Supplementary material for: Investigating microRNA Profiles in Prostate Cancer Bone Metastases and Functional Effects of microRNA-23c and microRNA-4328
Source: Cancers (Basel). 2023 Apr 24;15(9):2437. doi: 10.3390/cancers15092437 (PMC10177411; doi:10.3390/cancers15092437)
Supplement: Supplementary file 1 [file cancers-15-02437-s001.zip › Supplementary Table S3.pdf]

Table S3. Proposed functions of proteins significantly affected by stable overexpression of miRNA-23c or -4328 in 22Rv1 or PC-3 cells, according to proteomic profiling by LC-MS/MS. Only proteins predicted as theoretical targets for miRNA-23c or -4328 are shown (www.targetscan.org). Functions are listed as stated in the Universal Protein Resource Database (www.uniprot.org).

3A. Suggested functions of proteins significantly affected (adj*p* < 0.05) by overexpression of miRNA-23c 22Rv1. Downregulated proteins are shown in bold.

Abbreviation	Protein name	Function according to the Universal Protein Resource Database (direct citation)
**A1BG**	alpha-1-B glycoprotein	*Unknown*
**ERLIN2**	ER lipid raft associated 2	Involved in regulation of cellular cholesterol homeostasis by regulation the SREBP signalling pathway.
**RPIA**	ribose 5-phosphate isomerase A	Component of the pentose phosphate pathway.
**WBP2**	WW domain binding protein 2	Acts as transcriptional coactivator of oestrogen and progesterone receptors.
**YOD1**	YOD1 deubiquitinase	Participates in endoplasmic reticulum-associated degradation (ERAD) for misfolded luminal proteins.

3B. Suggested functions of proteins significantly affected (adj*p*<0.05) by miRNA-4328 overexpression in 22Rv1 cells. Downregulated proteins are shown in bold.

Abbreviation	Protein name	Function according to the Universal Protein Resource Database (direct citation)
**ADAM9**	ADAM metallopeptidase domain 9	Cleaves and releases a number of molecules with important roles in tumorigenesis and angiogenesis, such as TEK, KDR, EPHB4, CD40, VCAM1 and CDH5. May mediate cell-cell, cell-matrix interactions and regulate the motility of cells via interactions with integrins.
**ATG2A**	autophagy related 2A	Involved in autophagosome assembly, regulating the size of nascent autophagosomes. Tethers the edge of the isolation membrane (IM) to the endoplasmic reticulum (ER) and mediates direct lipid transfer from ER to IM for IM expansion.
**BARD1**	BRCA1 associated RING domain 1	The BRCA1-BARD1 heterodimer specifically mediates the formation of 'Lys-6'-linked polyubiquitin chains and coordinates a diverse range of cellular pathways such as DNA damage repair, ubiquitination and transcriptional regulation to maintain genomic stability.
**FMR1**	fragile X mental retardation 1	Plays a central role in neuronal development and synaptic plasticity through the regulation of alternative mRNA splicing, mRNA stability, mRNA dendritic transport and postsynaptic local protein synthesis of a subset of mRNAs
**ITIH4**	inter-alpha-trypsin inhibitor heavy chain family, member 4	Type II acute-phase protein (APP) involved in inflammatory responses to trauma.
**MFN1**	mitofusin 1	Mitochondrial outer membrane GTPase that mediates mitochondrial clustering and fusion
**PIKFYVE**	phosphoinositide kinase, FYVE finger containing	Dual specificity kinase implicated in myriad essential cellular processes such as maintenance of endomembrane homeostasis, and endocytic-vacuolar pathway, lysosomal trafficking, nuclear transport, stress- or hormone-induced signalling and cell cycle progression. Mediates EGFR trafficking to the nucleus.
**TRABD**	TraB domain containing	Unknown

3C. Suggested functions of proteins significantly affected (adj*p* < 0.05) by overexpression of miRNA-23c in PC-3 cells. Downregulated proteins are shown in bold.

Abbreviation	Protein name	Function according to the Universal Protein Resource Database (direct citation)
**APAF1**	apoptotic peptidase activating factor 1	Autocatalytic activation of pro-caspase-9, leading to the activation of caspase-3 and apoptosis.

**LMNB1**	lamin B1	Lamins are components of the nuclear lamina.
MED14	mediator complex subunit 14	Component of the Mediator complex, a coactivator involved in the regulated transcription of nearly all RNA polymerase II-dependent genes.
PJA1	praja ring finger 1, E3 ubiquitin protein ligase	Ubiquitinates Melanoma-associated antigen D1 (MAGED1) antigen leading to its subsequent degradation by proteasome. MAGED1 is involved in the apoptotic response after nerve growth factor (NGF) binding in neuronal cells. Inhibits cell cycle progression and facilitates NGFR-mediated apoptosis.
**STIM2**	stromal interaction molecule 2	Functions as a highly sensitive Ca^{2+} sensor in the endoplasmic reticulum which activates both store-operated and store-independent Ca^{2+}-influx.

3D. Suggested functions of proteins significantly affected (adj*p* < 0.05) by overexpression of miRNA-4328 in PC-3 cells. Downregulated proteins are shown in bold.

Abbreviation	Protein name	Function according to the Universal Protein Resource Database (direct citation)
**ART4**	ADP-ribosyltransferase 4	Catalytic protein mediating peptidyl-arginine ADP-ribosylation
**DIMT1**	DIM1 dimethyladenosine transferase 1 homolog	Specifically, dimethylates two adjacent adenosines in the loop of a conserved hairpin near the 3'-end of 18S rRNA in the 40S particle. Involved in the pre-rRNA processing steps leading to small-subunit rRNA production independently of its RNA-modifying catalytic activity.
**FERMT2**	fermitin family member 2	Enhances integrin-mediated cell adhesion onto the extracellular matrix and cell spreading. Plays a role in the TGFB1 and integrin signaling pathways. Plays a role in the regulation of transcription mediated by CTNNB1 and TCF7L2/TCF4 and in Wnt signalling.
**ITIH4**	inter-alpha-trypsin inhibitor heavy chain family, member 4	Type II acute-phase protein (APP) involved in inflammatory responses to trauma.
**PSMA4**	proteasome (prosome, macropain) subunit, alpha type, 4	Component of the 20S core proteasome complex involved in the proteolytic degradation of most intracellular proteins. This type of proteolysis is required in several pathways including spermatogenesis (20S-PA200 complex) or generation of a subset of MHC class I-presented antigenic peptides (20S-PA28 complex).
**UNC80**	unc-80 homolog	Component of the NALCN sodium channel complex, required for channel regulation. This complex is a cation channel activated by neuropeptides substance P, neurotensin, and extracellular calcium that regulates neuronal excitability by controlling the sizes of NALCN-dependent sodium-leak current.
